# Three new species of *Inosperma* (*Inocybaceae*, *Agaricales*) from western Sichuan, China, based on morphological and multigene phylogenetic evidence

**DOI:** 10.3897/mycokeys.141.200822

**Published:** 2026-09-25

**Authors:** Rui Wu, Peng-Ping Duan, Meng-Meng Lai, Zheng-Mi He, Zuo-Hong Chen

**Affiliations:** 1 Hunan Research Center of the Basic Discipline Developmental Biology, College of Life Sciences, Hunan Normal University, Changsha 410081, China Hunan Research Center of the Basic Discipline Developmental Biology, College of Life Sciences, Hunan Normal University Changsha China

**Keywords:** Diversity, new taxa, phylogeny, subalpine regions, taxonomy

## Abstract

Despite extensive mycological records from western Sichuan, China, no species of *Inosperma* have been reported from this area. In the present study, we describe three new *Inosperma* species from the subalpine regions of western Sichuan, namely, *I. fibrifugum*, *I. glomerisquamulosum*, and *I. lilacinifolium*. Phylogenetic analysis based on sequences of ITS, nrLSU, and *rpb2* indicates that these species form three distinct, strongly supported subclades. Specifically, *I. fibrifugum* and *I. glomerisquamulosum* fall within the Cervicolores clade, sister to *I. mucidiolens* and *I. turietoense*, respectively. *I. lilacinifolium* belongs to the Maculatum clade, clustering with *I. quietiodor* and *I. shawarense*. *Inosperma fibrifugum* is characterized by a fibrillose to scaly, fuscous brown pileus with scales persisting only on the umbo at maturity and a context that bruises reddish-brown. *Inosperma glomerisquamulosum* is characterized by small basidiomata with a yellowish-brown pileus covered with distinct scales, an indistinct odor, and the absence of caulocystidia. *Inosperma lilacinifolium* is characterized by a radially fibrillose, grayish-brown pileus and lilac lamellae, along with the presence of arachnoid veil remnants when young. A key to the Chinese species of *Inosperma* is provided.

## Introduction

*Inosperma* (Kühner) Matheny & Esteve-Rav., typified by *I. calamistratum* (Fr.) Matheny & Esteve-Rav., is one of the seven genera of the family *Inocybaceae* Jülich (Matheny et al. 2020). Originally established by Kühner (1980) as a subgenus of *Inocybe* s. str. to encompass smooth-spored *Inocybe* without pleurocystidia, *Inosperma* was re-evaluated based on molecular phylogenetic evidence. Matheny et al. (2020) demonstrated that *Inosperma* forms a well-supported monophyletic lineage distinct from *Inocybe*, warranting its elevation to genus rank to reflect its evolutionary independence.

*Inosperma* is characterized by a rimose to scaly pileus, a rubescent or brunnescent context, a distinct odor, phaseoliform spores, thin-walled cheilocystidia, and the absence of pleurocystidia (Kühner 1980; Matheny et al. 2020). *Inosperma* is ectomycorrhizal, forming symbiotic associations with vascular plants, predominantly with members of the families *Fagaceae*, *Pinaceae*, and *Salicaceae* (Kirk et al. 2008). Some species of *Inosperma* are reported to contain the neurotoxins muscarine, ibotenic acid, and muscimol (Kosentka et al. 2013; Deng et al. 2021a, 2021b; Li et al. 2022). Therefore, precise taxonomic identification is crucial for public health and the prevention of mushroom poisoning in China, as it serves as the foundation for distinguishing toxic species from edible ones. Phylogenetically, analyses by Larsson et al. (2009) and Kropp et al. (2013) suggested that section Cervicolores constitutes a monophyletic group. Building upon this foundation, recent molecular studies have resolved *Inosperma* into six stable lineages: the Cervicolores and Maculatum clades, the *I. misakaense* and *I. africanum* lineages, and two Old World Tropical clades (Larsson et al. 2009; Kropp et al. 2013; Pradeep et al. 2016; Matheny et al. 2020; Deng et al. 2021a).

Currently, the Index Fungorum online database lists 94 species names of *Inosperma* (http://www.indexfungorum.org; accessed 22 May 2026). These species are distributed in Africa, Asia, Australasia, Europe, and North America (Matheny et al. 2020). Among these, 19 *Inosperma* species have been reported from the northeastern, northwestern, southwestern, and southernmost regions of China (Fan and Bau 2018; Deng et al. 2021a, 2021b, 2022; Li et al. 2022; Zhou et al. 2023; Liu et al. 2026). Western Sichuan, China, situated at the intersection of the Hengduan Mountains and the southeastern margin of the Qinghai-Tibet Plateau, features complex topography and climate that sustain rich fungal diversity (Ying et al. 1994; Cui et al. 2016, 2018; He et al. 2023; Gao et al. 2024). This ecological setting is particularly significant, as the dominant vegetation includes widespread populations of potential host plants, particularly members of the genera *Abies*, *Betula*, *Picea*, *Pinus*, *Taxus*, *Larix*, *Rhododendron*, *Salix*, and *Quercus* (He 2009), creating a favorable environment for *Inosperma* diversity. However, at present, there are no records of *Inosperma* species from this region. In this study, we first describe three new species of *Inosperma* from this region, based on morphological and multi-locus phylogenetic evidence.

## Materials and methods

### Specimens

Fresh basidiomata were photographed in their natural habitat in western Sichuan, China. Collection details were recorded, and the specimens were transported to the laboratory for further processing. Fresh basidiomata were dried at 45 °C (Tarafder et al. 2025, 2026). These specimens are deposited in the Mycological Herbarium of Hunan Normal University (MHHNU, Changsha, China). Detailed information about these specimens, including the species name, location, and the herbarium and GenBank numbers, is provided in Table 1.

**Table 1.** Sequences used in phylogenetic analysis.

**Species**	**Voucher**	**Locality**	**ITS**	**LSU**	***RPB2***	**References**
*Auritella arenicolens*	OKM 23824 (PERTH)	Australia	MF474176	MF476239	MF490438	Matheny et al. (2017)
*A. brunnescens*	NLB 942 (holotype)	Australia	KJ702342	KJ702339	KJ702347	Matheny et al. (2017)
*Inosperma acutofulvum*	MCVE 29416	Italy	MG944832	–	–	Bizio et al. (2017)
*I. adaequatum*	JV 11290F	Finland	JQ801381	JQ815407	AY333771	Matheny et al. (2020)
*I. africanum*	MR 00387	Togo	MN096189	MN097881	MT770739	Aïgnon et al. (2021)
*I. africanum*	HLA 0383 (holotype)	Benin	MT534298	MT560733	–	Aïgnon et al. (2021)
*I. afromelliolens*	PC 96013	Zambia	JQ801383	EU600883	EU600882	Pradeep et al. (2016)
*I. akirnum*	CAL 1358 (holotype)	India	KY440085	KY549115	KY553236	Matheny et al. (2020)
*I. apiosmotum*	PBM 2784 (TENN 062582)	USA	JQ801384	EU555454	EU555453	Kropp et al., unpublished
*I. apollonium*	SMNS-STU-F-0901670 (holotype)	Germany	NR_184499	ON003428	–	Bandini et al. (2021)
*I. apricum*	STU: SMNS-STU-F-0901844	Germany	PP962258	PP962258	–	Eberhardt, unpublished
*I. bicoloratum*	ZT 12187	Malaysia	GQ892984	GQ892938	JQ846464	Pradeep et al. (2016)
*I. boeticum*	PC 96082	Zambia	JQ801412	JN975027	–	Ryberg and Matheny (2012)
*I. bongardii*	EL 9406	Sweden	FN550943	FN550943	–	Aïgnon et al. (2023)
*I. bulbomarginatum*	MR 00357 (holotype)	Benin	MN096190	MN097882	MN200775	Aïgnon et al. (2021)
*I. calamistratoides*	PBM 3384 (TENN 065750)	Australia	JQ801393	JQ815415	KJ729949	Kropp et al. (2013); Matheny and Bougher (2017)
*I. aff. calamistratum*	REH 8420	Costa Rica	JQ801390	JN975018	JQ846471	Pradeep et al. (2016)
*I. carnosibulbosum*	TBGT 12047	India	KT329448	KT329454	KT329443	Pradeep et al. (2016)
*I. cervicolor*	TURA 4761	Finland	JQ801395	JQ815417	JQ846474	Pradeep et al. (2016)
*I. changbaiense*	HMJAU 25861 (holotype)	China	NR_160610	NG_066407	MT086755	Fan and Bau (2018)
*I. confusum*	GDOR 5575 (holotype)	Italy	PX289195	PX289307	PX317238	Crous et al. (2026)
*I. cookei*	EL 70A03	Sweden	AM882953	AM882953	–	Ryberg et al. (2008)
*I. cyanotrichium*	PERTH 08516952	Australia	JQ801396	JN975033	JQ846476	Matheny and Bougher (2017)
*I. erubescens*	JV 9070F	Finland	–	EU569846	–	Pradeep et al. (2016)
*I. aff. fastigiellum*	JRH 408 (WTU)	USA	–	AY380374	AY333770	Matheny (2005)
*I. aff. fastigiellum*	PBM 3325	USA	JQ801399	JQ815419	JQ846477	Pradeep et al. (2016)
***I. fibrifugum***	MHHNU 34360 (holotype)	China	**PZ384882**	**PZ389101**	**PZ403746**	**This study**
***I. fibrifugum***	MHHNU 34361	China	**PZ384883**	**PZ389102**	**PZ403747**	**This study**
*I. flavobrunneum*	HLA 0746	Benin	OQ446450	OQ293897	OQ435247	Aïgnon et al. (2023)
*I. friesii*	GB-0207737 (holotype)	Sweden	PV962265	PV962265	–	Crous et al. (2026)
*I. friesii*	AH 15648	Spain	PV989624	PV989625	–	Crous et al. (2026)
*I. fulvum*	EL 166-08	Sweden	FJ904171	–	–	Larsson et al. (2009)
*I. geminum*	JV 31497 (TUR) (holotype)	Sweden	OR823936	OR823936	PP092174	Esteve-Raventós et al. (2024)
*I. geraniodorum*	EL 106-06 (GB 0207617)	Sweden	FN550945	FN550945	–	Pradeep et al. (2016)
***I. glomerisquamulosum***	MHHNU 35000 (holotype)	China	**PZ384885**	**PZ389105**	**PZ403750**	**This study**
***I. glomerisquamulosum***	MHHNU 34456	China	**PZ384884**	**PZ389103**	**PZ403748**	**This study**
***I. glomerisquamulosum***	MHHNU 34870	China	**PZ384886**	**PZ389104**	**PZ403749**	**This study**
*I. gracilentum*	EL 85-19(GB 0207620) (holotype)	Sweden	OR817726	OR817726	–	Esteve-Raventós et al. (2024)
*I. gregarium*	CAL 1309 (holotype)	India	KX852305	KX852306	KX852307	Latha and Manimohan (2017)
*I. hainanense*	Zeng 4937 (holotype)	China	MZ374070	MZ374761	MZ388104	Deng et al. (2021a)
*I. latericium*	TR 109-02 (M)	Papua New Guinea	JQ801405	JN975023	JQ846487	Pradeep et al. (2016)
*I. lijiangense*	GMB-W 1237 (holotype)	China	PX589498	PX589500	–	Liu et al. (2026)
*I. lijiangense*	GMB-W 1238	China	PX589499	PX589501	–	Liu et al. (2026)
***I. lilacinifolium***	MHHNU 34956 (holotype)	China	**PZ387120**	**PZ389109**	**PZ403754**	**This study**
***I. lilacinifolium***	MHHNU 34951	China	**PZ387117**	**PZ389106**	**PZ403751**	**This study**
***I. lilacinifolium***	MHHNU 34953	China	**PZ387118**	**PZ389107**	**PZ403752**	**This study**
***I. lilacinifolium***	MHHNU 34954	China	**PZ387119**	**PZ389108**	**PZ403753**	**This study**
***I. lilacinifolium***	MHHNU 34957	China	**PZ387121**	**PZ389110**	**PZ403755**	**This study**
*I. longisporum*	MHNNU 32337 (holotype)	China	OP135509	OP135495	OP161560	Li et al. (2022)
*I. macrocarpa*	HLA 0791 (holotype)	Benin	OQ300373	OQ300370	OQ435245	Aïgnon et al. (2023)
*I. maculatum*	EL 74-05	Sweden	AM882959	–	–	Ryberg et al. (2008)
*I. aff. maculatum*	PBM 2446	USA	DQ241778	AY745700	EU569863	Pradeep et al. (2016)
*I. maximum*	PBM 2222/UBC F33244	USA/Canada	MG953983	EU569854	–	Pradeep et al. (2016)
*I. misakaense*	PC 96234	Zambia	JQ801409	EU569875	AY333767	Pradeep et al. (2016)
*I. mucidiolens*	DG 1824 (holotype)	Canada	HQ201339	HQ201340	–	Pradeep et al. (2016)
*I. mucidiolens*	WTU: F-043330	USA	NR119899	–	–	Schoch et al. (2014)
*I. muscarium*	FYG 6091 (holotype)	China	MZ373982	MZ373991	MZ388093	Deng et al. (2021a)
*I. mutatum*	PBM 4108, PBM 2542	USA	MG773837	AY732212	DQ472729	Matheny et al. (2020)
*I. neobrunnescens*	PBM 2452	USA	–	EU569868	EU569867	Matheny and Moreau (2009)
I. neobrunnescens var. leucothelotum	SAT0427406	USA	JQ801411	JN975025	JQ846489	Pradeep et al. (2016)
*I. neohirsutum*	EL 163-15 (GB 0207661)	Sweden	OR831119	OR831119	PP092169	Esteve-Raventós et al. (2024)
*I. nivalellum*	MHNNU3 1689 (holotype)	China	OP135502	OP134006	OP161556	Li et al. (2022)
*I. pakistanicum*	LAH 38334 (holotype)	Pakistan	PQ452108	PQ614170	–	Sana et al. (2026)
*I. pallidifolium*	PBM 2451	USA	JQ408759	JQ319690	JQ846483	Pradeep et al. (2016)
*I. pallidifolium*	TENN-F-021175 (isotype)	USA	OP162983	–	–	Eberhardt et al. (2023)
*I. pavithrum*	DKP-SERB 65 (CALI)	India	PP350421	PP192110	PP209642	Crous et al. (2026)
*I. praetermissum*	PBM 1105	USA	JQ801386	JQ815409	JQ846466	Pradeep et al. (2016)
*I. praetermissum*	ACAD: 10206	Canada	MH024863	MH539758	–	Matheny et al., unpublished
*I. praetermissum*	EL 161-18 (GB 0243037)	Sweden	OR831120	OR831120	PP092170	Esteve-Raventós et al. (2024)
*I. proximum*	ZT 13015	Thailand	EU600839	EU600840	–	Matheny et al. (2020)
*I. quietiodor*	EL 115_04	Sweden	AM882960	AM882960	–	Pradeep et al. (2016)
*I. quietiodor*	PAM 01091310	France	FJ936168	FJ936168	–	Larsson et al. (2009)
*I. cf. reisneri*	G 0928/LAS 97067	Hungary/Sweden	AM882974	MK278242	–	Ryberg et al., unpublished
*I. rhodiolum*	EL 223_06	France	FJ904175	FJ904175	–	Pradeep et al. (2016)
*I. rimosoides*	PBM 2459	USA	DQ404391	AY702014	DQ385884	Pradeep et al. (2016)
*I. rosellicaulare*	ACAD: 11618 (holotype)	Canada	MT237482	–	–	Matheny et al., unpublished
*I. rubricosum*	PBM 3784 (TENN)	Australia	KP308817	KP170990	KM406230	Pradeep et al. (2016)
*I. saragum*	CAL 1360	India	KY440103	KY549133	KY553249	Latha and Manimohan (2017)
*I. shawarense*	ASSW79A (holotype)	Pakistan	KY616964	KY616966	–	Naseer et al. (2018)
*I. shawarense*	FLAS-FS9456	Pakistan	KY616965	–	–	Naseer et al. (2018)
*I. sphaerobulbosum*	MHNNU 32266 (holotype)	China	OP135501	OP134001	OP161559	Li et al. (2022)
*I. squamulosobrunneum*	MHNNU 32359 (holotype)	China	OP135499	OP134000	OP161562	Li et al. (2022)
*I. squamulosohinnuleum*	MHNNU 32195 (holotype)	China	OP135500	OP134002	OP161558	Li et al. (2022)
*I. sorongense*	BO 24735 (holotype)	Indonesia	PQ687483	PQ687484	–	Retnowati et al. (2026)
*I. subhirsutum*	EL 198-18 (GB 0243041)	Sweden	OR817730	OR817730	PP092164	Esteve-Raventós et al. (2024)
*I. submaculatum*	PC 96073	Zambia	JQ801417	EU600870	EU600869	Pradeep et al. (2016)
*I. subinodorum*	MCVE 21578 (isotype)	Italy	JF908231	–	–	Crous et al. (2026)
*I. subrubescens*	HRL 0948/F-2989	USA	KX897436	PQ652584	–	Matheny et al., unpublished
*I. subsphaerosporum*	FYG 5848 (FHMU3153) (holotype)	China	MW403825	MW397171	MW404237	Deng et al. (2021b)
*I. turietoense*	AH 47710 (holotype)	Spain	PP431526	PP431541	PP478208	Esteve-Raventós et al. (2024)
*I. turietoense*	AH 47669	Spain	PP431525	PP431540	–	Esteve-Raventós et al. (2024)
*I. veliferum*	EB 1993080906	Italy	PP431519	PP431539	–	Esteve-Raventós et al. (2024)
*I. vinaceobrunneum*	PBM 2951 (TENN 062709) (holotype)	USA	FJ601813	HQ201353	JQ846478	Matheny and Kudzma (2019)
*I. vinaceum*	AMB 18747	Italy	MW561108	MW561120	–	Cervini et al. (2021)
*I. viridipes*	PBM 3767(holotype)	Australia	NR_153168	KP171094	KM656138	Latha and Manimohan (2017)
*I. virosum*	TBGT 753(holotype)	India	KT329452	KT329458	KT329446	Pradeep et al. (2016))
*I. wuzhishanense*	FYG 7352	China	OR436485	OR436491	OR451204	Zhou et al. (2023)
*I. zonativeliferum*	FYG 6441 (holotype)	China	OL850878	OM845772	ON075044	Deng et al. (2022)

Note: Newly generated sequences are shown in bold, and “-” represents data unavailability.

### Morphological analysis

Macroscopic characters were described based on field records. The color codes were based on Kornerup and Wanscher (1978). The size categories followed the standards of Bas (1969) and Yang (1997), with basidiomata classified into five grades according to pileus diameter: very small (diameter ≤ 3 cm), small (3–5 cm), medium (5–9 cm), large (9–15 cm), and very large (≥ 15 cm). Dry specimens were mounted on glass slides, rehydrated in a 5% KOH solution, and, when necessary, stained with a 1% Congo red solution to facilitate the observation of microscopic structures. Basidiospores, basidia, cystidia, lamellae trama, pileipellis and stipitipellis were observed using a Nikon Eclipse 50i microscope (Nikon, Tokyo, Japan). Side-views of mature basidiospores were measured, with a minimum of 20 spores analyzed from each of the basidiomata. The sampling scope is denoted as [n/m/p], representing n basidiospores measured from m basidiomata of p collections. Dimensional data are presented in the format (a) b–e–c(d), where e represents the mean, b and c define the boundaries of the central 90% of the measured values, and a and d represent the extreme minimum and maximum values, respectively. Xm represents the mean length of basidiospores ± sample standard deviation × mean width of basidiospores ± sample standard deviation. Q means the length/width ratio of a basidiospore, and Qm represents the mean Q of all basidiospores ± standard deviation. For scanning electron microscopy (SEM), a small fragment of the lamella was excised from the dried specimen using forceps and affixed to a metallic specimen stub using double-sided adhesive tape. The sample was then coated with a thin layer of gold (Au) to prevent charging (Tarafder et al. 2022, 2023). Micrographs of the basidiospores were captured under high vacuum conditions at various magnifications. This work was performed using a JSM-IT800 field-emission microscope (JEOL, Tokyo, Japan) at the Central South University Science Park, Changsha, China.

### DNA extraction, PCR amplification, and sequencing

Genomic DNA was extracted using a fungal DNA extraction kit from Omega Bio-Tek (Norcross, GA, USA). The following primer pairs were used for PCR amplification and sequencing: ITS1F and ITS4 were used for the internal transcribed spacer (ITS) (White et al. 1990; Gardes and Bruns 1993); LR0R and LR5 (Vilgalys and Hester 1990) for the 28S nuclear ribosomal large subunit (nrLSU); and bRPB2-6F and bRPB2-7.1R for the RNA polymerase II second largest subunit (*rpb2*) (Matheny 2005). PCR was performed according to the protocols described by White et al. (1990) for ITS and nrLSU and Matheny (2005) for *rpb2*, using Kodaq 2× PCR MasterMix (Tolo Biotechnology Co., Ltd., Shanghai, China). PCR products were sent to Zhejiang Youkang Biotechnology Co., Ltd (Huzhou, China) for sequencing.

### Phylogenetic analysis

SeqMan v. 6.01 (Swindell and Plasterer 1997) and BioEdit v. 7.0.9 (Hall 1999) were used to remove low-quality regions from the sequences. Publicly available sequences from previous studies by Pradeep et al. (2016), Latha and Manimohan (2017), Matheny and Bougher (2017), Fan and Bau (2018), Matheny et al. (2020), Aïgnon et al. (2021), Bandini et al. (2021), Deng et al. (2021a, 2021b, 2022), Li et al. (2022), and Liu et al. (2026) were retrieved from the GenBank database (https://www.ncbi.nlm.nih.gov/genbank/; accessed 21 May 2026). *Auritella arenicolens* (Cleland) Matheny & Bougher (OKM 23824) and *A. brunnescens* Matheny & Bougher (NLB 942) were selected as outgroups (Matheny et al. 2017). A total of 247 sequences (95 ITS, 90 nrLSU, and 62 *rpb2*) were used, of which 30 sequences (10 ITS, 10 nrLSU, and 10 *rpb2*) were newly generated (Table 1).

Sequences were aligned using MAFFT v. 7.130b (Katoh and Standley 2013). AliView v. 1.23 (Larsson 2014) was used for subsequent manual adjustments to ensure the accuracy of the alignments. The concatenated ITS-nrLSU-*rpb2* dataset was generated using PhyloSuite v. 1.2.2 (Zhang et al. 2020). The dataset consisted of 502 bp of ITS, 885 bp of nrLSU, and 731 bp of *rpb2* from 84 taxa. The best-fit nucleotide substitution models for each partition were determined using MrModeltest v. 2.3 (Nylander 2004), with GTR+I+G selected for all partitions based on the Akaike information criterion (AIC). Bayesian inference (BI) analysis was conducted using MrBayes v. 3.2.7a (Ronquist et al. 2012) using the selected models. The analysis involved two parallel runs of 10,000,000 generations, during which trees were sampled every 100 generations. The initial 25% of sampled trees were discarded as burn-in, and convergence was confirmed by ensuring that the average standard deviation of split frequencies was below 0.01. Maximum likelihood (ML) analysis was performed in RAxML v. 8.2.12 (Stamatakis 2014) using the GTR+I+G model, with branch support assessed by 1,000 bootstrap replicates. Trees were edited and formatted using FigTree v. 1.4.3 (Rambaut 2018) and Adobe Illustrator CS3 v.11.0.2 (https://www.adobe.com).

## Results

### Phylogenetic analysis

**Figure 1.**
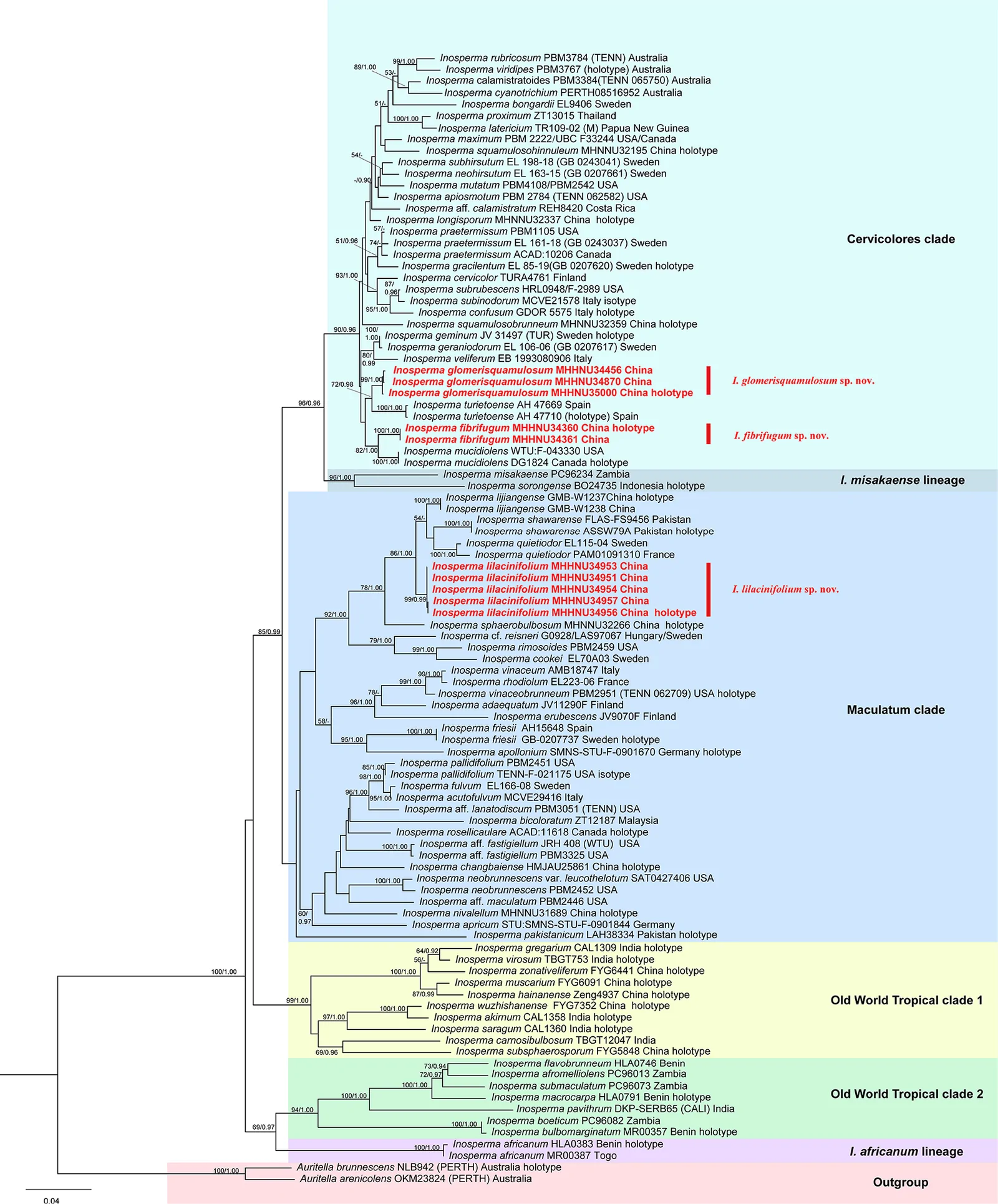
Phylogenetic tree of *Inosperma* inferred from maximum likelihood (ML) and Bayesian inference (BI) analyses of the combined dataset (ITS, nrLSU, and *rpb2*). Maximum likelihood bootstrap values (MLBS) > 50% and Bayesian posterior probabilities (BPP) > 0.90 are shown at the nodes (MLBS/BPP). The new species are highlighted in red.

The topologies of the phylogenetic trees obtained from the ML and BI analyses were congruent. The phylogram resulting from the ML analysis is shown in Fig. 1, with Maximum likelihood bootstrap (MLBS) and Bayesian posterior probability (BPP) values labeled on the branches. With the genus *Auritella* used as an outgroup, *Inosperma* forms a fully supported monophyletic lineage (MLBS = 100%, BPP = 1.00). *Inosperma* is further resolved into six lineages: (1) the Cervicolores clade (MLBS = 90%, BPP = 0.96), (2) the *I. misakaense* lineage (MLBS = 96%, BPP = 1.00), (3) the Maculatum clade (with low support), (4) Old World Tropical clade 1 (MLBS = 99%, BPP = 1.00), (5) Old World Tropical clade 2 (MLBS = 94%, BPP = 1.00), and (6) the *I. africanum* lineage (MLB = 100%, BPP = 1.00). *Inosperma fibrifugum* (MLBS = 100%, BPP = 1.00) and *I. glomerisquamulosum* (MLBS = 99%, BPP = 1.00) fall within the Cervicolores clade, while *I. lilacinifolium* (MLBS = 99%, BPP = 0.99) is nested within the Maculatum clade. *I. fibrifugum* is sister to *I. mucidiolens* within the Cervicolores clade, with strong support (MLBS = 82%, BPP = 1.00); *I. glomerisquamulosum* is recovered as sister to *I. turietoense* (MLBS = 72%, BPP = 0.98). *I. lilacinifolium* forms a distinct clade, which is sister to the clade containing *I. quietiodor*, *I. shawarense*, and *I. lijiangense* (MLBS = 86%, BPP = 1.00).

### Taxonomy

#### Inosperma fibrifugum

Taxon classificationFungiAgaricalesInocybaceae

R. Wu, Z.M. He & Z.H. Chen sp. nov.

36D1968F-583B-5F34-A99C-0641972C3A13

Fungal Names: FN 573830

Fig. 2

##### Chinese Name.

脱纤鳞歧盖伞 (Pinyin: tuō xiān lín qí gài săn).

**Figure 2.**
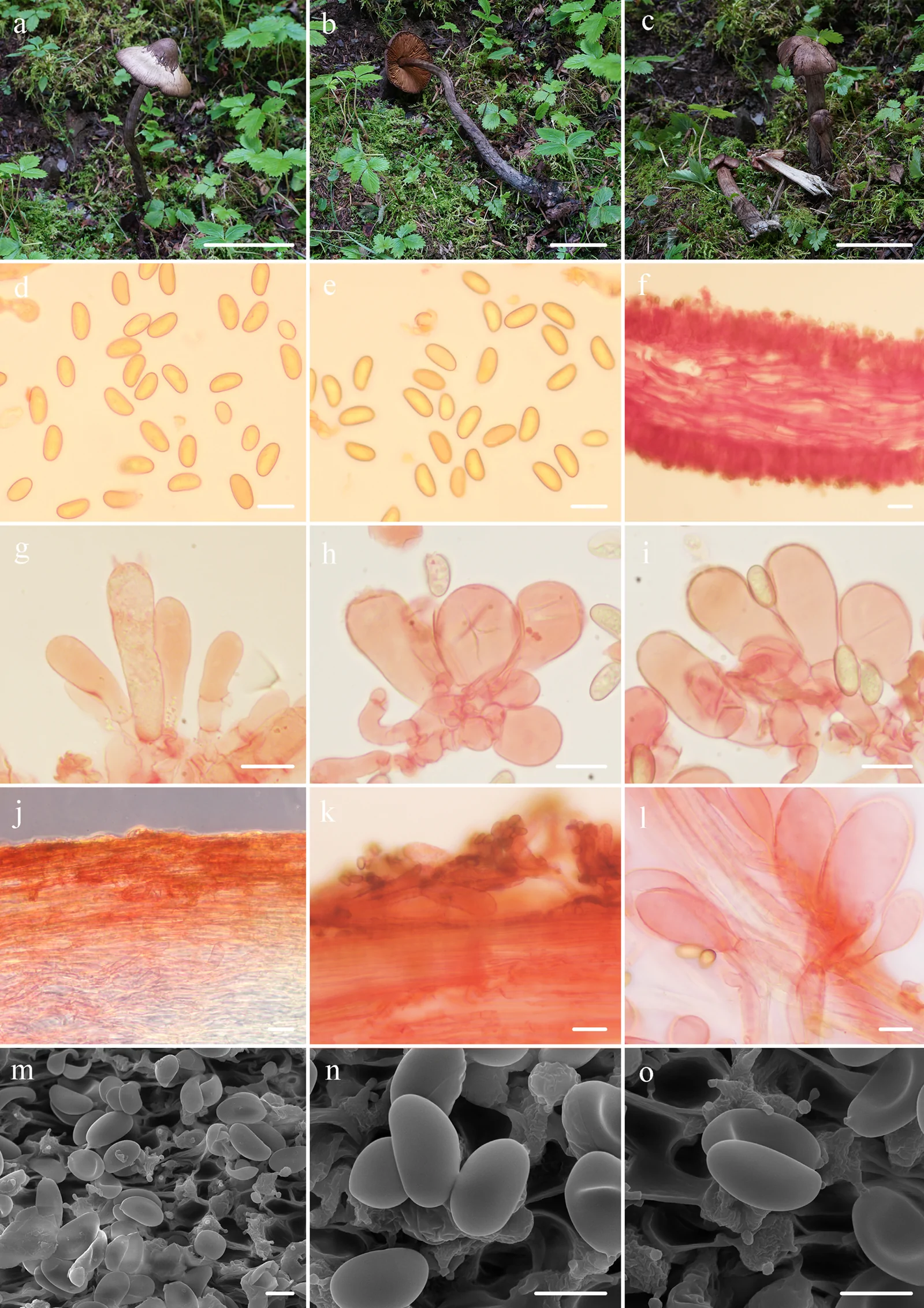
Morphological features of *Inosperma fibrifugum* (MHHNU 34360, holotype). **a, b, c**. Basidiomata (**a, b**. MHHNU 34360; **c**. MHHNU 34361); **d, e**. Basidiospores; **f**. Lamellae trama; **g**. Basidia; **h, i**. Cheilocystidia; **j**. Pileipellis; **k**. Stipitipellis; **l**. Caulocystidia; **m, n, o**. Basidiospores under SEM. Scale bars: 5 cm (**a–c**); 10 μm (**d, e, g–i, l**); 20 μm (**f, j, k**); 5 μm (**m–o**).

##### Etymology.

The specific epithet “*fibrifugum*” refers to the fibrillose scales at the margin of the pileus that slough off with age.

##### Diagnosis.

Similar to *I. mucidiolens* phylogenetically, but differs in having larger basidiomata, pileus scales persisting only on the umbo at maturity, narrower cheilocystidia, larger caulocystidia, and lacking the odor of fresh corn.

##### Type.

China • Sichuan Province, Xiaojin County, Mengbi Mountain, in a subalpine forest with trees of *Pinus* spp., *Picea* spp. and *Camellia* spp., at 31°43.01'N, 102°16.76'E, alt. 3,685 m, 2 Aug. 2024, Z.H. Chen MHHNU 34360 (**holotype**).

##### Description.

***Basidiomata*** very small to medium-sized. ***Pileus*** 16–52 mm in diam, at first hemispherical, later becoming campanulate with an obtusely umbonate center, surface dry, dark brown (5E3–5) around center, elsewhere grayish white (2B1–2) to whitish brown (5C2–3), radially fibrillose to scaly; scales appressed to erect, brownish (5E5–7) to brown (6E4–6), gradually slough off at the margin when mature; margin incurved to plane, sometimes with faint, brownish black (5D4–5), radially rimose striae; context dirty white (1A1) to creamy white (1A2–3). ***Context*** up to 2 mm thick, becoming dark brown (6E7–8) to reddish-brown (7E6–8) when bruised. ***Lamellae*** 1–3 mm wide, adnate, crowded, unequal, initially cream white (1A2–3) to yellowish white (2A2–3), becoming brown (6E4–5) to dark brown (5E5–7) with age, reddish-brown (8D6–8) when bruised; edges undate to undulate, brownish white (4B2–3) to yellowish (3A2–4). ***Stipe*** 44–151 × 5–13 mm, subcylindrical central, solid when young, becoming fistulose in the lower part when mature; surface fibrillose to tomentose, yellowish brown (5D6–8) to brown (5E3–6) at apex and in the middle, bluish-green (25D5–7) to indigo (24C4–6) at base; context grayish white (2B1) to cream white (3A1). ***Context*** of stipe becoming dark brown (6E7–8) to reddish-brown (7E6–8) when bruised. ***Odor*** indistinct.

***Basidiospores*** [62/2/2] (9.0–)10.0–12.0(–12.5) × (4.5–)5.0–6.5(–7.0) µm, Xm = 11.0 ± 0.75 × 5.8 ± 0.43, Q = (1.87–)1.88–1.95(–1.96), Qm = 1.92 ± 0.02, ellipsoid to oblong, yellowish brown in 5% KOH, hyaline, thin-walled, smooth. ***Basidia*** 32–41 × 7–12 µm, clavate, 4-spored, thin-walled, hyaline in 5% KOH, sometimes with yellowish brown contents; sterigmata 2–5 µm long. ***Lamellae trama*** subregular, composed of thin-walled, hyaline, 4–11 um wide hyphae. ***Pleurocystidia*** absent. ***Cheilocystidia*** 21–38 × 11–19 µm, clavate to broadly clavate, occasionally obovoid, colorless, hyaline, thin-walled, with yellowish-brown pigment on the wall. ***Pileipellis*** a cutis composed of cylindrical thin-walled hyphae 3–12 µm wide, with encrusting brown pigment, with scattered raised terminal elements 62–79 × 9–12 µm. ***Subpellis*** composed of subfusiform, fusiform to cylindrical colorless hyphae 2–15 µm wide. ***Stipitipellis*** composed of longitudinally arranged, 2–9 µm wide, hyphae, colorless, hyaline, thin-walled, with encrusting brown pigment. ***Caulocystidia*** 29–39 × 12–17 µm, clavate to broadly clavate. ***Clamps*** present in all parts of hyphae.

##### Ecology.

Scattered, on soil, in subalpine forests with trees of *Pinus* spp., *Picea* spp., and *Camellia* spp.

##### Distribution.

Currently known from southwestern China.

##### Additional specimen examined.

China • Sichuan Province, Xiaojin County, Mengbi Mountain, in a subalpine forest with trees of *Pinus* spp., *Picea* spp. and *Camellia* spp., at 31°43.01'N, 102°16.76'E, alt. 3,685 m, 2 Aug. 2024, Z.H. Chen, MHHNU 34361 (Paratype).

##### Notes.

*Inosperma fibrifugum* is characterized by a pileus surface covered with fibrillose, appressed scales when young; these scales persist only at the umbo upon maturation. Its context turns reddish-brown when bruised. *Inosperma fibrifugum* forms a sister species pair with *I. mucidiolens* (Grund & Stuntz) Matheny & Esteve-Rav. (Fig. 1). In both species, the stipe and lamellae become reddish when bruised, and the basidiospores and basidia have similar dimensions. However, *I. mucidiolens* has shorter stipes (30–90 mm), pileus scales that do not slough off at maturity, longer cheilocystidia (33–50 µm), larger caulocystidia (27–50 × 10–22 µm), and an odor reminiscent of fresh corn (Grund and Stuntz 1970).

*Inosperma glomerisquamulosum*, a newly described species in this study, is closely related to *I. fibrifugum*. Both species share a hemispherical to campanulate pileus and similar basidiospore shapes. However, *I. glomerisquamulosum* is distinguished by its smaller basidiomata (4–45 mm), lack of reddening when bruised, stipe base lacking bluish-green to indigo coloration, larger basidiospores (13.0–15.5 × 6.5–8.0 µm vs. 10.0–12.0 × 5.0–6.5 µm), and absence of caulocystidia.

*Inosperma cervicolor* (Pers.) Matheny & Esteve-Rav. shares with *I. fibrifugum* a centrally scaled, conic-campanulate pileus, context and lamellae that bruise reddish-brown, and a grayish-green stipe base. However, *I. cervicolor* is distinguished by its smaller basidiomata (pileus 6–40 mm), larger basidiospores (10.5–14.5 × 6.5–8.0 µm vs. 10.0–12.0 × 5.0–6.5 µm), and larger cheilocystidia (35–6 × 9–15 µm). Furthermore, it has a strong, disagreeable odor resembling that of old wine casks (Kuyper 1986).

#### Inosperma glomerisquamulosum

Taxon classificationFungiAgaricalesInocybaceae

R. Wu, Z.M. He & Z.H. Chen sp. nov.

56B36D11-C85D-5AA2-9126-2A01DC50EB4F

Fungal Names: FN 573831

Fig. 3

##### Chinese Name.

簇鳞歧盖伞 (Pinyin: cù lín qí gài săn).

**Figure 3.**
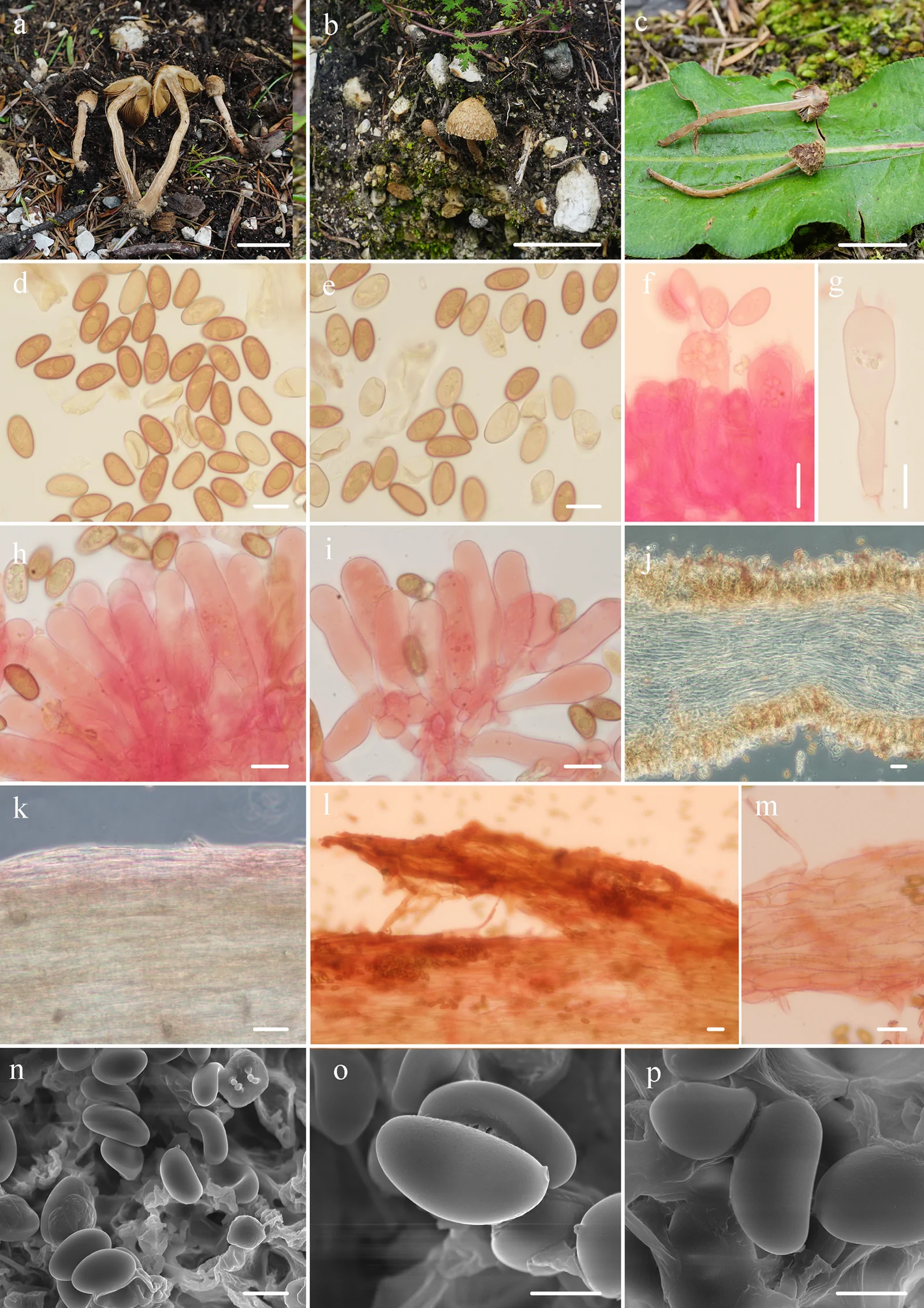
Morphological features of *Inosperma glomerisquamulosum* (MHHNU 35000, holotype). **a, b, c**. Basidiomata (**a, b**. MHHNU 35000; **c**. MHHNU 34870); **d, e**. Basidiospores; **f, g**. Basidia; **h, i**. Cheilocystidia; **j**. Lamellae trama; **k**. Stipitipellis; **l, m**. Pileipellis and hyphae; **n, o, p**. Basidiospores under SEM. Scale bars: 5 cm (**a–c**); 10 μm (**d–i, m**); 20 μm (**j–l**); 5 μm (**n–p**).

##### Etymology.

“*glomerisquamulosum*” refers to the clustered scales on the pileus.

##### Diagnosis.

Closely affiliated with *I. turietoense*, but differs by larger basidiomata, a stipe that turns dark brown upon touch, a context that does not change color when bruised, larger basidiospores, the absence of caulocystidia, and a distinctive odor.

##### Type.

China • Sichuan Province, Daocheng County, Daocheng Yading Scenic Area, in a subalpine forest with trees of *Quercus aquifolioides*, Larix potaninii var. macrocarpa, Picea likiangensis var. rubescens, *Rhododendron* spp., *Salix* spp. and *Rosa* spp., at 28°26.05'N, 100°21.34'E, alt. 3,930 m, 9 Aug. 2025, Z.H. Chen, MHHNU 35000 (**holotype**).

##### Description.

***Basidiomata*** very small to small. ***Pileus*** 4–45 mm in diam., hemispherical to paraboloid, lacks a distinct umbo when young, becoming campanulate and slightly umbonate with age; surface dry, yellowish brown (4A4–5) to dark brown (5D5–7), covered with clustered and subimbricate appressed scales yellowish white (3A1–3) to yellowish brown (4A4–5) when young; erect conical scales dark brown (5D5–7) at the center, outer region fibrillose, scales occasionally reflexed, at maturity; margin initially incurved, becoming plane to slightly reflexed and rimose. ***Context*** up to 3 mm thick, whitish brown (3B2–3) when young, turning yellowish (3B5–7) to brownish (5F6–8) when mature. ***Lamellae*** 1–2 mm wide, adnexed to adnate, crowded, unequal, initially dirty white (1A1) to gray-brown (4A2–4), becoming yellowish-brown (4B5–7), dark brown (5E6–8) to fuscous (5F7–8) at maturity; edges serrate to undulate, dirty white (1A1–2) to grayish (1C1–3). ***Stipe*** 22–106 × 1–4 mm, subcylindrical, central, solid, often slightly broader at base; surface covered with yellowish-brown (2B4–6) to rust-brown (3D5–7) fibrillose scales and floccose tomentum, background brownish white (2A1–3) to yellowish-brown (3C3–5), turning dark brown (5E6–8) upon touched, occasionally with sparse arachnoid fibrils at apex; context yellowish-brown (3B4–6) to brownish (5E5–6). ***Odor*** indistinct.

***Basidiospores*** [163/3/3] (12.0–)13.0–15.5(–17.0) × (6.0–)6.5–8.0(–9.0) µm, Xm = 14.2 ± 0.82 × 7.4 ± 0.56, Q = (1.81–)1.86–1.97(–1.99), Qm = 1.93 ± 0.04, elongate, brownish in 5% KOH, hyaline, thin-walled, smooth. ***Basidia*** 45–54 × 10–14 µm, clavate, 4-spored, thin-walled, hyaline, colorless to yellowish in 5% KOH, sometimes with sparse yellowish-brown oily drops; sterigmata 2–7 µm long. ***Lamellae trama*** subregular, composed of thin-walled, hyaline, 3–13 um wide hyphae. ***Pleurocystidia*** absent. ***Cheilocystidia*** 28–56 × 8–15 µm, narrowly clavate, occasionally narrowly utriform, colorless, hyaline, thin-walled. ***Pileipellis*** a cutis composed of cylindrical, slightly inflated to inflated, thin-walled hyphae, 3–12 µm wide, with brown encrusting pigment. ***Subpellis*** composed of subfusiform, fusiform, to cylindrical colorless hyphae, 8–24 µm wide. ***Stipitipellis*** composed of brown to yellowish brown, thin-walled hyphae, 11–32 µm wide, walls smooth to sparsely encrusted. ***Caulocystidia*** absent. ***Clamps*** present in all parts of hyphae.

##### Ecology.

Scattered, on soil, in subalpine forests with trees of *Larix potaninii*, *Picea asperata* and *Ribes vilmorinii*.

##### Distribution.

Known from Southwestern China.

##### Additional specimens examined.

China • Sichuan Province, Songpan County, Munigou, in a subalpine forest with trees of *Picea asperata* and *Ribes vilmorinii*, at 32°33.90'N, 103°30.25'E, alt. 3,120 m, 8 Aug. 2025, Z.H. Chen MHHNU 34456 (Paratype); • Xiaojin County, Siguniangshan, Shuangqiaogou, in a coniferous forest dominated by *Larix potaninii*, at 31°14.74'N, 102°46.91'E, alt. 3,830 m, 29 Jul. 2025, Z.H. Chen MHHNU 34870 (Paratype).

##### Notes.

*Inosperma glomerisquamulosum* is characterized by small basidiomata. Its pileus surface is covered with appressed, clustered, yellowish-brown scales when young. At maturity, there are erect conical scales in the center, and the outer clusters of appressed scales change from appressed-fibrillose to fibrillose. Caulocystidia are absent in this species, and its odor is indistinct. *Inosperma glomerisquamulosum* is phylogenetically closely related to *I. turietoense* Pancorbo & Esteve-Rav. (Fig. 1). Both species possess hemispherical pileus with appressed scales on their surfaces and have smooth, ellipsoid basidiospores. Their basidia and cheilocystidia are also comparable in shape and size. However, *I. turietoense* has smaller basidiomata (pileus 20–30 mm), a context that turns reddish upon bruising, and smaller basidiospores (10.4–13.4 × 6.4–7.3 μm vs 13.0–15.5 × 6.5–8.0 µm) (Esteve-Raventós et al. 2024). This species also possesses caulocystidia and has a distinct odor, ranging from earthy to moldy and sometimes blended with aromatic components (Esteve-Raventós et al. 2024).

*Inosperma glomerisquamulosum* is morphologically similar to *I. gracilentum* E. Larss. & Esteve-Rav., sharing a conical-campanulate pileus and a stipe apex covered with minute scales. However, *I. gracilentum* exhibits smaller basidiomata (pileus 5–25 mm), reddening of the upper stipe context with a bluish-green base, and smaller basidiospores (10.8–13.7 × 6.0–7.4 μm vs. 13.0–15.5 × 6.5–8.0 µm), as well as the presence of caulocystidia (20–42 × 9–16 μm) and a distinct odor of pelargonium or fish, distinguishing it from *I. glomerisquamulosum* (Esteve-Raventós et al. 2024).

Both *Inosperma glomerisquamulosum* and *I. neohirsutum* Esteve-Rav. et al. possess a conical-campanulate pileus with erect conical scales at the center at maturity. Their basidia and cheilocystidia are of the same size and shape. However, *I. neohirsutum* differs in having smaller basidiomata (pileus 15–25 mm). The upper part of the stipe turns slightly reddish when bruised. It has a bluish green stipe base, smaller basidiospores (9.0–10.9 × 5.0–6.1 μm vs. 13.0–15.5 × 6.5–8.0 µm), caulocystidia and a variable odor. This odor can be faint, aromatic, or fruity-acidic at times, and at other times, it may have a scent of pelargonium or fish (Esteve-Raventós et al. 2024).

#### Inosperma lilacinifolium

Taxon classificationFungiAgaricalesInocybaceae

R. Wu, Z.M. He & Z.H. Chen sp. nov.

678F89F1-AC96-5AE2-8CEC-7E3C477FB02A

Fungal Names: FN 573832

Fig. 4

##### Chinese Name.

淡紫褶歧盖伞 (Pinyin: dàn zĬ zhě qí gài săn).

**Figure 4.**
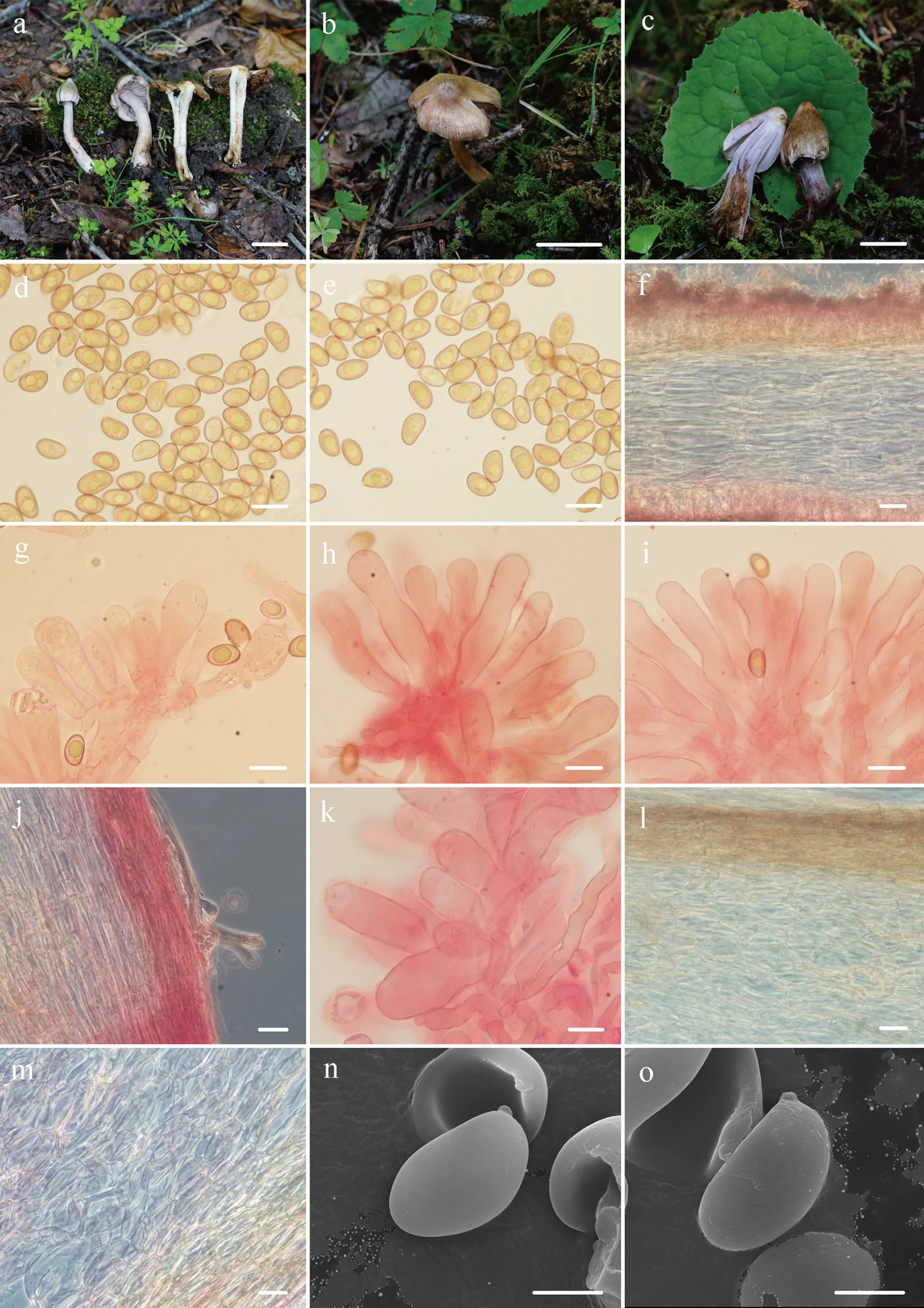
Morphological features of *Inosperma lilacinifolium* (MHHNU 34956, holotype). **a, b, c**. Basidiomata (**a**. MHHNU 34956; **b**. MHHNU 34951; **c**. MHHNU 34953); **d, e**. Basidiospores; **f**. Lamellae trama; **g**. Basidia; **h, i**. Cheilocystidia; **j**. Stipitipellis; **k**. Caulocystidia; **l, m**. Pileipellis and hyphae; **n, o**. Basidiospores under SEM. Scale bars: 2 cm (**a–c**); 10 μm (**d, e, g–i, k**); 20 μm (**f, j, l, m**); 5 μm (**n, o**).

##### Etymology.

“*lilacinifolium*” refers to the grayish-purple lamellae observed in young basidiomata.

##### Diagnosis.

Similar to *I. quietiodor*, but differs in having the distinct pileus coloration, a more prominent umbo, the presence of arachnoid veil remnants in its youth, larger basidiospores, cheilocystidia and caulocystidia, and the lack of a flour-like odor resembling that of *Lactarius quietus* or a special farinaceous odor.

##### Type.

China • Sichuan Province, Jiuzhaigou County, Shenxianchi Scenic Area, in a subalpine forest with trees of *Abies* spp., *Salix* spp., *Juniperus* spp., *Betula* spp. and *Malus* spp., at 33°22.58'N, 103°41.05'E, alt. 2,905 m, 3 Aug. 2025, Z.H. Chen MHHNU 34956 (**holotype**).

##### Description.

***Basidiomata*** very small. ***Pileus*** 10–30 mm in diam., conical to campanulate when young, then convex to plano-convex with age, with a distinct umbo at center, becoming broadly umbonate when mature, surface dry, grayish (1A1), gray (1B1–2) to gray-brown (1C2–3), radially fibrillose, often covered with a thin coating of dirty white (2B1–2) veil remnants at center when young; margin at first slightly incurved to decurved, later reflexed and often splited. ***Context*** up to 2 mm thick, white (1A1), sometimes yellow (1B6–8) to brown (5D3–5) under the pileal center. ***Lamellae*** up to 2 mm wide, adnexed, crowded, initially dirty white (18A1–2) to dusty purple (18B1–3), becoming grayish brown (1B2), brown (5D3–5) to dark brown (5F5–7) when old, with unequal lamellae, becoming attenuate towards stipe; edges fimbriate, grayish (2B1) to gray (3B1–2). ***Stipe*** 35–40 × 2–8 mm, central, solid, subcylindrical to cylindrical, often slightly inflated at base; surface dirty white (2A1) to brownish white (2C2–3), pruinose, occasionally with sparse arachnoid fibrils near the apex of stipe, longitudinally striate downward the stipe, basal tomentum present, white (1A1) to brownish (5C2–3). ***Context*** white (1A1), sometimes yellow (1B6–8) to brown (5D3–5) in the middle of the stipe. ***Odor*** indistinct.

***Basidiospores*** [133/4/4] (8.0–)9.0–11.0(–12.0) × (5.0–)5.5–7.0(–7.5) µm, Xm = 9.9 ± 0.71 × 6.1 ± 0.53, Q = (1.54–)1.56–1.66(–1.67), Qm = 1.63 ± 0.03, ellipsoid to elongate, yellowish-brown in 5% KOH, hyaline, thin-walled, smooth. ***Basidia*** 29–34 × 9–14 µm, clavate, 4-spored, thin-walled, hyaline, colorless to yellowish in 5% KOH; sterigmata 3–5.5 µm long. ***Lamellae trama*** subregular, composed of thin-walled, hyaline, 5–21 um wide hyphae. ***Pleurocystidia*** absent. ***Cheilocystidia*** 23–61 × 8–14 µm, narrowly clavate, slender, colorless, hyaline, thin-walled. ***Pileipellis*** a cutis composed of filamentous, thin-walled hyphae 3–12 µm wide, with intracellular and encrusting brown pigment. ***Subpellis*** composed of subfusiform, fusiform to cylindrical, colorless hyphae 8–24 µm wide and filamentous hyphae 3–8 µm wide. ***Stipitipellis*** composed of hyaline filamentous hyphae 4–10 µm wide. ***Caulocystidia*** 33–69 × 8–13 µm, clavate, colorless, hyaline, thin- to slightly thick-walled. ***Clamps*** present in all parts of hyphae.

##### Ecology.

Scattered, on soil, in subalpine forests with trees of *Abies* spp., *Salix* spp., *Juniperus* spp., *Betula* spp., and *Malus* spp.

**Distribution**. Known from southwestern China.

##### Additional specimens examined.

China • Sichuan Province, Jiuzhaigou County, Shenxianchi Scenic Area, in a subalpine forest with trees of *Abies* spp., *Salix* spp., *Juniperus* spp., *Betula* spp. and *Malus* spp., at 33°22.58'N, 103°41.05'E, alt. 2,928 m, 3 Aug. 2025, Z.H. Chen MHHNU 34951 (Paratype); • same location and date, Z.H. Chen MHHNU 34953 (Paratype); • same location and date, Z.H. Chen MHHNU 34954 (Paratype); same location and date, Z.H. Chen MHHNU 34957 (Paratype).

##### Notes.

*Inosperma lilacinifolium* is characterized by very small basidiomata, a pileus with a distinct umbo, a gray to gray-brown color, a radially fibrillose surface, and frequently present dirty-white veil remnants. Its lamellae exhibit a unique grayish-purple tint when young, turning brown at maturity. Its stipe is silvery-white with arachnoid veil remnants near the apex, and it has large, ellipsoid, thin-walled basidiospores. Its odor is indistinct.

*Inosperma lilacinifolium* is phylogenetically closely related to *I. shawarense* (Naseer & Khalid) Aïgnon & Naseer (Fig. 1). Although the two species share similar basidiomata sizes, conical to campanulate pilei, fibrillose surfaces, and indistinct odors, *I. shawarense* is distinguished by its brownish-white pileus with a rimose surface cracking toward the margin and lacking veil remnants, as well as by its smaller, thick-walled, phaseoliform basidiospores (4.7–6.5 × 2.8–3.7 μm), smaller cheilocystidia (21–55 × 9–11 μm) and caulocystidia (21–55 × 9–11 μm), and a pileipellis with blunt-ended hyphae lacking encrusting brown pigment (Naseer et al. 2018).

*Inosperma quietiodor* (Bon) Matheny & Esteve-Rav. and *I. lilacinifolium* have basidiomata of similar size. Their basidiomata are characterized by a radially fibrillose, umbonate pileus, a fibrillose stipe, and comparably sized basidia. However, *I. quietiodor* differs in having an ochraceous buff to yellow pileus, a lower and broader umbo, smaller basidiospores (8.0–10.0 × 5.0–6.0 µm vs. 9.0–11.0 × 5.5–7.0 µm), smaller cheilocystidia (28–58 × 10–17 µm) and caulocystidia (28–58 × 10–17 µm), and a peculiar odor resembling that of *Lactarius quietus* or a farinaceous odor (Kuyper 1986).

*Inosperma pakistanicum* Sana & Kiran, a vividly colored species from Pakistan, shows similarities to *I. lilacinifolium*. It has a conical to campanulate pileus with a central umbo and rimose margin at maturity, violet-tinted lamellae when young, and a slightly inflated stipe base. The two species exhibit comparable dimensions and shapes of basidiospores. However, *I. pakistanicum* is distinguished by its larger basidiomata (pileus 30–58 mm), ochraceous to orange pileus, purple lamellae that turn reddish-orange at maturity, smaller basidia (22.49–27.91 × 7.28–8.35 µm), narrower cheilocystidia (6.84–7.90 µm), and the absence of caulocystidia (Kiran and Ali 2025).

### Key to the *Inosperma* species reported in China

**Table:** 

1	Pileus not smooth, tomentose or coarsely fibrillose, or with appressed, squarrose to erect scales; basidia slender	**2**
–	Pileus smooth, rimulose or rimose; basidia not slender	**7**
2	Caulocystidia absent; not discoloring when bruised; odor indistinct; pileus covered with clustered and subimbricate appressed scales	***I. glomerisquamulosum***
–	Caulocystidia present; discoloring when bruised; odor distinct	**3**
3	Stipe base without bluish-green tinge; odor strong, disagreeable, musty, like old wine casks	***I. cervicolor***
–	Stipe base with bluish-green tinge	**4**
4	Spores oblong-ellipsoid (Qm > 2); without veil remnants	**5**
–	Spores ellipsoid (Qm ≤ 2); with veil remnants	**6**
5	Basidiospores (9.5–)10.0–13.5(–14.0) × 5.0–6.0(–6.5) µm; discoloring pinkish or pale vinaceous when bruised; odor faint, pelargonium-like with a faintly fishy note	***I. calamistratum***
–	Basidiospores (9.0–)12.0–17.0 × (5.0–)5.5–7.0(–8.0) µm; discoloring brown when bruised; odor faintly maize-like in young basidiomata, becoming strongly spermatic in mature specimens	***I. longisporum***
6	Pileus surface covered with erect fibrils, conical and scattered around the center; cheilocystidia densely arranged in grape-like clusters; turning brown when bruised	***I. squamulosobrunneum***
–	Pileus surface with small, granular, erect scales; cheilocystidia in clusters; turning red when bruised	***I. squamulosohinnuleum***
7	Context discolors when bruised	**8**
–	Context does not discolor when bruised	**12**
8	Pileus with veil remnants; caulocystidia absent	**9**
–	Pileus without veil remnants; caulocystidia present	**10**
9	Context brownish white to pale pinkish when bruised; odor indistinct	***I. wuzhishanense***
–	Context yellowish brown to olive green when bruised; odor fungoid or grassy	***I. zonativeliferum***
10	Pileus 16–52 mm, with fibrillose scales disappearing at maturity, remaining only at center; odor indistinct	***I. fibrifugum***
–	Pileus broadly expanded (≥ 60 mm in diameter)); odor strongly pungent	**11**
11	Odor sweetish and disagreeable, soap-like; context staining orange-red to red-brown; basidiospores (9.5–)10.0–14.5(–15.0) × (5.5–)6.0–7.5(–8.0) µm	***I. erubescens***
–	Odor spermatic, of rotting acorns, green corn, or sickly-sweetish; discoloring red-brown on bruising; basidiospores 9.0–12.5(–14.0) × 5.5–7.0(–7.5) µm	***I. adaequatum***
12	Caulocystidia absent	**13**
–	Caulocystidia present	**16**
13	Pileus with veil remnants	**14**
–	Pileus without veil remnants	**15**
14	Basidiospores (5.0–)6–7.5(–8.0) × 5.0–6.0(–6.5) μm; odor slightly salty or mild; stipe apex recurved-squamulose	***I. subsphaerosporum***
–	Basidiospores 8.0–10.0(–11.0) × 5.0–6.0(–6.5) µm; odor fungoid, slightly grassy or mild; Stipe apex with sparse fibrils, longitudinally fibrillose downward	***I. muscarium***
15	Cheilocystidia (29.63–)32.84–42.05(–44.61) × (9.18–)9.65–12.06(–12.89) μm; stipe surface fibrous; odor not recorded	***I. lijiangense***
–	Cheilocystidia 34–55 × 15–25 µm; stipe smooth, surface longitudinally striate; odor indistinct or slightly acid	***I. hainanense***
16	Pileus with veil remnants	**17**
–	Pileus without veil remnants, or not recorded	**18**
17	Odor spermatic; stipe apex with scattered pallid furfuraceous fibrils; basidiospores (6.5–)7.0–10.5(–11.0) × (4.5–)5.0–5.5(–6.0) µm	***I. sphaerobulbosum***
–	Odor indistinct; Stipe pruinose and striate; with sparse apical fibrils and basal tomentum; basidiospores (8.0–)9.0–11.0(–12.0) × (5.0–)5.5–7.0(–7.5) µm	***I. lilacinifolium***
18	Stipe base non-inflated	***I. nivalellum***
–	Stipe base inflated	**19**
19	Pileus hemispheric to plano-expanded, umbo obtuse or indistinct; odor resembling rape leaf juice	***I. quietiodor***
–	Pileus conical to applanate, with prominent umbo; odor conspicuous	**20**
20	Lamellae grayish-white to yellowish-brown; odor earthy	***I. lanatodiscum***
–	Lamellae white to grayish, tinged olivaceous at maturity; odor strongly spermatic	***I. changbaiense***

## Discussion

Through morphological and multilocus phylogenetic analyses (ITS, nrLSU, and *rpb2*), this study describes three new *Inosperma* species from western Sichuan, China. This study therefore enriches the known species diversity of the genus and fills the gap in *Inosperma* records from western Sichuan. These three species are characterized by a rimose-to-scaly pileus, phaseoliform spores, thin-walled cheilocystidia, and the absence of pleurocystidia, thereby conforming to the generic characteristics of *Inosperma* (Kühner 1980; Matheny et al. 2020). *Inosperma glomerisquamulosum* lacks caulocystidia. *Inosperma lilacinifolium* has a radially fibrillose, grayish-brown pileus, lilac lamellae, and sparse arachnoid fibrils near the apex of the stipe. *Inosperma glomerisquamulosum* and *I. lilacinifolium* lack a distinct odor, and their contexts do not discolor upon bruising. These characteristics distinguish them from other species in the genus.

In line with recent phylogenetic studies (Larsson et al. 2009; Kropp et al. 2013; Pradeep et al. 2016), our phylogenetic analysis recognizes six major lineages within *Inosperma* s. str., including the Cervicolores and Maculatum clades, the *I. misakaense* and *I. africanum* lineages, and two Old World Tropical clades (Larsson et al. 2009; Kropp et al. 2013; Pradeep et al. 2016; Deng et al. 2021a). These lineages are strongly supported (MLBS = 90–100%, BPP = 0.96–1.00), except for the Maculatum clade, which receives poor statistical support (MLBS < 50%, BPP < 0.90). Notably, recently described species from China are concentrated in the Cervicolores, Maculatum, and Old World Tropical 1 clades (Fan and Bau 2018; Deng et al. 2021a, 2021b, 2022; Li et al. 2022; Zhou et al. 2023; Liu et al. 2026). The new species *I. fibrifugum* (MLBS = 100%, BPP = 1.00) and *I. glomerisquamulosum* (MLBS = 99%, BPP = 1.00) are classified within the Cervicolores clade. This clade is typically characterized by species showing pronounced reddening of the context, a fibrillose, woolly, tomentose, scaly, or strigose pileus, and very slender (Q > 4.0), necropigmented basidia (Matheny et al. 2020; Esteve-Raventós et al. 2024). The morphological features of *I. fibrifugum* and *I. glomerisquamulosum* are largely consistent with that definition. However, *I. glomerisquamulosum* exhibits no reddening of the context. Thus, the Cervicolores clade contains species that redden upon bruising as well as species that do not change color. *Inosperma lilacinifolium* belongs to the Maculatum clade, which is typically characterized by little or no reddening, a smooth and radially fibrous to rimose pileus, and basidia that lack necropigment and are not as slender (Q < 4.0) (Matheny et al. 2020; Esteve-Raventós et al. 2024). The context of *I. lilacinifolium* shows almost no reddening; its stipe turns dark brown when touched. The presence of a filamentous veil when young and of arachnoid veil remnants at the stipe apex at maturity serves as a distinguishing feature of this species.

## Supplementary Material

XML Treatment for Inosperma fibrifugum

XML Treatment for Inosperma glomerisquamulosum

XML Treatment for Inosperma lilacinifolium

## Additional information

### Artificial Intelligence (AI) use

The authors accept full responsibility for the content of the manuscript, including the disclosure of any use of AI.

No AI tools were used in the preparation of this manuscript.

### Author ORCIDs

R. Wu https://orcid.org/0000-0002-8187-5241

P.-P. Duan https://orcid.org/0009-0005-1142-7944

M.-M. Lai https://orcid.org/0009-0004-6519-6654

Z.-M. He https://orcid.org/0000-0001-8754-3427

Z.-H. Chen https://orcid.org/0000-0001-7359-0257

### Data availability

The DNA sequences obtained in this study have been submitted to GenBank.
